# Analysis of the serial circulating tumor cell count during neoadjuvant chemotherapy in breast cancer patients

**DOI:** 10.1038/s41598-020-74577-w

**Published:** 2020-10-15

**Authors:** Sungchan Gwark, Jisun Kim, Nak-Jung Kwon, Kyoung-Yeon Kim, YongNam Kim, Cham Han Lee, Young Hun Kim, Myoung Shin Kim, Sung Woo Hong, Mi Young Choi, Byung Hee Jeon, Suhwan Chang, Jonghan Yu, Ji Yeon Park, Hee Jin Lee, Sae Byul Lee, Il Yong Chung, Beom Seok Ko, Hee Jeong Kim, Jong Won Lee, Byung Ho Son, Jin-Hee Ahn, Kyung Hae Jung, Sung-Bae Kim, Gyung-Yub Gong, Sei Hyun Ahn

**Affiliations:** 1 Department of Surgery, University of Ulsan, College of Medicine, Asan Medical Center, Seoul, Korea; 2 Macrogen Inc, Seoul, Korea; 3 Cytogen Inc, Seoul, Korea; 4 Department of Biomedical Sciences, University of Ulsan, College of Medicine, Asan Medical Center, Seoul, Korea; 5 Department of Surgery, Division of Breast and Endocrine Surgery, Sungkyunkwan University School of Medicine, Samsung Medical Center, Seoul, Korea; 6 Department of Pathology, University of Ulsan, College of Medicine, Asan Medical Center, Seoul, Korea; 7 Department of Oncology, University of Ulsan, College of Medicine, Asan Medical Center, Seoul, Korea

**Keywords:** Breast cancer, Tumour biomarkers, Breast cancer

## Abstract

We evaluated the prognostic implications of the circulating tumor cell (CTC) count in non-metastatic, HER2-negative breast cancer patients who failed to achieve pathologic complete response (pCR) after neoadjuvant chemotherapy (NCT). A total of 173, non-metastatic breast cancer patients treated with NCT were prospectively enrolled. CTCs were obtained from blood drawn pre-NCT and post-NCT using a SMART BIOPSY SYSTEM isolation kit (Cytogen Inc., Seoul, Korea) with immunofluorescence staining. Excluding 26 HER2-positive patients, Relapse-free survival (RFS) and overall survival (OS) related to the CTC count and the association of the CTC count with the treatment response to given therapy were analyzed in 147 HER2-negative patients. Among 147 HER2-negative patients, 28 relapses (19.0%) and 13 deaths (8.8%, all breast cancer-specific) were observed during a median follow-up of 37.3 months. One hundred and seven patients (72.8%) were hormone receptor-positive, and 40 patients (27.2%) had triple-negative breast cancer (TNBC). One or more CTCs were identified in 88 of the 147 patients (59.9%) before NCT and 77 of the 134 patients (52.4%) after NCT. In the entire HER2-negative patient cohort, the initial nodal status was the most significant factor influencing RFS and OS. In TNBC, 11 patients (27.5%) achieved pCR and patients that failed to achieve pCR with ≥ 5 CTCs after NCT, showed worse RFS (HR, 10.66; 95% CI, 1.80–63.07; *p* = 0.009) and OS (HR, 14.00; 95% CI, 1.26–155.53; *p* = 0.032). The patients with residual tumor and a high number of the CTCs after NCT displayed the worse outcome. These findings could provide justification to launch a future, well designed trial with longer follow-up data to obtain regulatory approval for clinical use of the assay, especially for the ER-positive, HER2-negative breast cancer subset.

## Introduction

Neoadjuvant chemotherapy (NCT) is becoming a more likely treatment of choice for locally advanced breast cancer patients. Down-staging could lead to a lower extent of surgery, e.g. an increase in the breast conservation rate, and a better cosmetic outcome^1–3^. Moreover, NCT allows in vivo monitoring of tumor response evaluation, thereby enabling prediction of the pathological response^4–9^.

However, patients who fail to achieve pathologic complete response (pCR) after NCT have poor prognoses^10^. Adjuvant anti-human epidermal growth factor receptor 2 (HER2) therapy is a reliable treatment option for HER2-positive breast cancer patients, especially for those with residual tumor burden^11^. In HER2-negative patients, trials evaluated the role of capecitabine as an adjuvant treatment, showed conflicting results. Masuda et al.^12^ showed HER2-negative patients with residual disease after NCT may benefit from adjuvant chemotherapy and with greater benefit, particularly among triple-negative breast cancer patients (TNBC). In a randomized trial by Coalición Iberoamericana de Investigación en Oncología Mamaria (CIBOMA/2004-01/GEICAM/2003-11; ClinicalTrials.gov number, NCT00130533)^13^, which evaluated the efficacy of adjuvant capecitabine after chemotherapy in TNBC patients, no significant survival benefit was observed. However, in subgroup analysis with non-basal like patients, significant benefit from capecitabine was found. While it is crucial to select the patients, who will truly benefit from additional adjuvant treatment, no such prognostic biomarker still exists.

Circulating tumor cells (CTCs) are cells that have been shed from the primary tumors or even from metastases, invade the blood vessels, and circulate in the bloodstream. CTCs can be the seeds that act as a cause of metastasis and such a hypothesis was first proposed by the Australian pathologist, Thomas Ashworth, in 1869^14^. These tumor cells in the bloodstream can be obtained via a simple blood draw, referred to as a ‘liquid biopsy’, and thus enabling less invasive and simpler assessment compared to tissue biopsies such as core needle biopsy or bone marrow aspiration^15^. Analyzing CTCs may offer more reliable and more direct information that can be used for monitoring the response to therapy, for selecting proper treatment agents, and as a potential biomarker of prognosis, yet most of the evidence is in regard to metastatic/treatment-resistant cancers. In metastatic breast cancer, CTC counts and their changes during treatment are well-known to be related to a poor prognosis^16,17^. Alternatively, conflicting results have been reported in several recent studies analyzing CTCs in non-metastatic patients with different types of cancers^18–22^. However, in breast cancer treated with NCT, not many researchers have investigated CTCs in relation to the treatment response and prognosis and in which only a small number of cases were analyzed^23–25^_._

The aim of the study is to address the clinical question whether CTC count could provide additional prognostic information besides pCR after NCT, which may guide to decide additional adjuvant chemotherapy. However, the use of neoadjuvant anti-HER2 regimen in South Korea is very complex regarding the insurance issue so the patients revealed a profound level of heterogeneity in terms of a given treatment. The authors decided to focus on HER2-negative breast cancer patients as done in CREATE-X trial from Masuda group^12^. Thus, we evaluated the prognostic implication of serial analysis of the CTC count in non-metastatic, HER2-negative, operable breast cancer patients who failed to achieve pCR after NCT.

## Results

### Baseline characteristics

The patient characteristics of the entire HER2-negative patient cohort and each subgroup are summarized in Table 1. Among 147 HER2-negative patients, 107 patients (72.8%) were hormone receptor-positive and 40 patients (27.2%) had TNBC. The mean age of the entire HER2-negative cohort was 45.8 years (range, 28 to 71 years; median age, 45 years). Sixty-nine of 147 HER2-negative patients underwent breast-conserving surgery followed by adjuvant radiation (100%) and 78 patients had a mastectomy. Of those 78 patients, 46 patients (59.0%) selectively received adjuvant radiation according to their condition (Tumor stage** ≥ **3 or Nodal stage** ≥ **2**)**. Ninety-three of 107 ER-positive, HER2-negative patients received tamoxifen (compliance rate 90.3%) and 14 patients received an aromatase inhibitor (compliance rate 92.9%) after surgery.

**Table 1:** Patient characteristics and the NCT regimen.

Variables	HER2-negative (n = 147)		ER-positive, HER2-negative (n = 107)		Triple-negative (n = 40)	
**Patient age**	Mean 45.81 (28–71)		Mean 45.48 (28–71)		Mean 46.21 (34–59)	
34 ≥	7	4.8%	6	5.6%	1	2.5%
35–50	106	72.1%	78	72.9%	28	70.0%
51 ≤	34	23.1%	23	21.5%	11	27.5%
**Type of surgery**						
Breast-conserving (adjuvant radiation)	69 (69)	46.9% (100.0%)	46 (46)	43.0% (100.0%)	23 (23)	57.5% (100.0%)
Mastectomy (adjuvant radiation)	78 (46)	53.1% (59.0%)	61 (35)	57.0% (57.4%)	17 (11)	42.5% (64.7%)
**Tumor subtype (pre-NCT)**						
ER-positive, HER2-negative	107	72.8%	107	100.0%	0	0%
Triple-negative	40	27.2%	0	0%	40	100%
**Clinical T stage**						
T1	15	10.2%	13	12.1%	1	5.0%
T2	96	65.3%	68	63.6%	28	70.0%
T3	34	23.1%	324	22.4%	10	25.0%
T4	2	1.4%	2	1.9%	0	0%
**Lymph node status**						
Negative	45	30.6%	34	31.8%	11	27.5%
Positive	102	69.4%	73	68.2%	29	72.5%
**Histologic grade**						
G1	2	1.4%	2	1.9%	0	0%
G2	109	74.1%	92	86.0%	17	42.5%
G3	34	23.1%	12	11.2%	22	55.0%
Unknown	2	1.4%	1	0.9%	1	2.5%
**ER status**						
Positive	107	72.8%	107	100.0%	0	0%
Negative	40	27.2%	0	0%	43	100%
**PR status**						
Positive	90	61.2%	90	84.1%	0	0%
Negative	57	38.8%	17	15.9%	43	100%
**Endocrine therapy**						
Tamoxifen (compliance)	N/A due to heterogenous population		93 (84)	86.9% (90.3%)	No endocrine therapy	
Aromatase inhibitor (compliance)			14 (13)	13.1% (92.9%)		
**Baseline CA-15-3**						
Normal	139	94.6%	103	96.3%	36	90.0%
Elevated	5	3.4%	2	1.9%	3	7.5%
Unknown	3	2.0%	2	1.9%	1	2.5%
**RECIST status (post-NCT)**						
Responder (CR + PR)	130	88.4%	98	91.6%	32	80.0%
Non-responder (SD + PD)	16	10.9%	9	8.4%	7	17.5%
Unknown	1	0.7%	0	0%	1	2.5%
**Pathologic response**						
pCR	18	12.2%	7	6.5%	11	27.5%
non-pCR	129	87.8%	100	93.5%	29	72.5%
**Neoadjuvant chemotherapy regimen (entire Her2-negative cohort, n = 147)**						
AC#4-					44(29.9%)	
AC#4 > D#4-					83(56.5%)	
FEC#4 > D#4-					14(9.5%)	
NCT02441933* (AC#4 > D + carboplatin#4)-					2(1.4%)	
NCT02032277* (veliparib/placebo + carboplatin/placebo + paclitaxel)-					4(2.7%)	

*AC* adriamycin and cyclophosphamide, *CR* complete response, *D* docetaxel, *FEC* fluorouracil, epirubicin, and cyclophosphamide, *pCR* pathologic complete response, *PD* progressive disease, *PR* partial response, *RECIST* response evaluation criteria in solid tumors, *SD* stable disease.

*The results of both trials have not yet been reported.

### Breast cancer cell recovery rate in spike-in test

To evaluate the cell recovery rate of the SMART BIOPSY SYSTEM Isolation kit (cat no. CIKW10; Cytogen, Inc., Seoul, Korea), a spike‑in test using MCF7 and MDA-MB231 cells was performed (100 cells each). Experiments were performed in triplicate. The average identified cell recovery rate of MCF7 and MDA-MB231 was 70.19% and 60.08%, respectively (Supplementary Figure S1 online).

### Treatment response and CTC count

All of the 147 HER2-negative patients underwent chemotherapy with either anthracycline or taxane or both (Table 1). One hundred thirty patients (88.4%, 130/147) of the patients showed a partial or complete response (PR or CR), and 16 patients (10.9%, 16/147) showed stable or progressive disease according to RECIST criteria^26^. Eighteen (12.2%, 18/147) patients achieved pCR. In subgroup analysis, the TNBC patients demonstrated a higher pCR rate (27.5%, 11/40), which is significantly higher than the hormone receptor-positive patients (6.5%, 7/107), as is well-known^27,28^ (Table 1).

**Figure 1:**
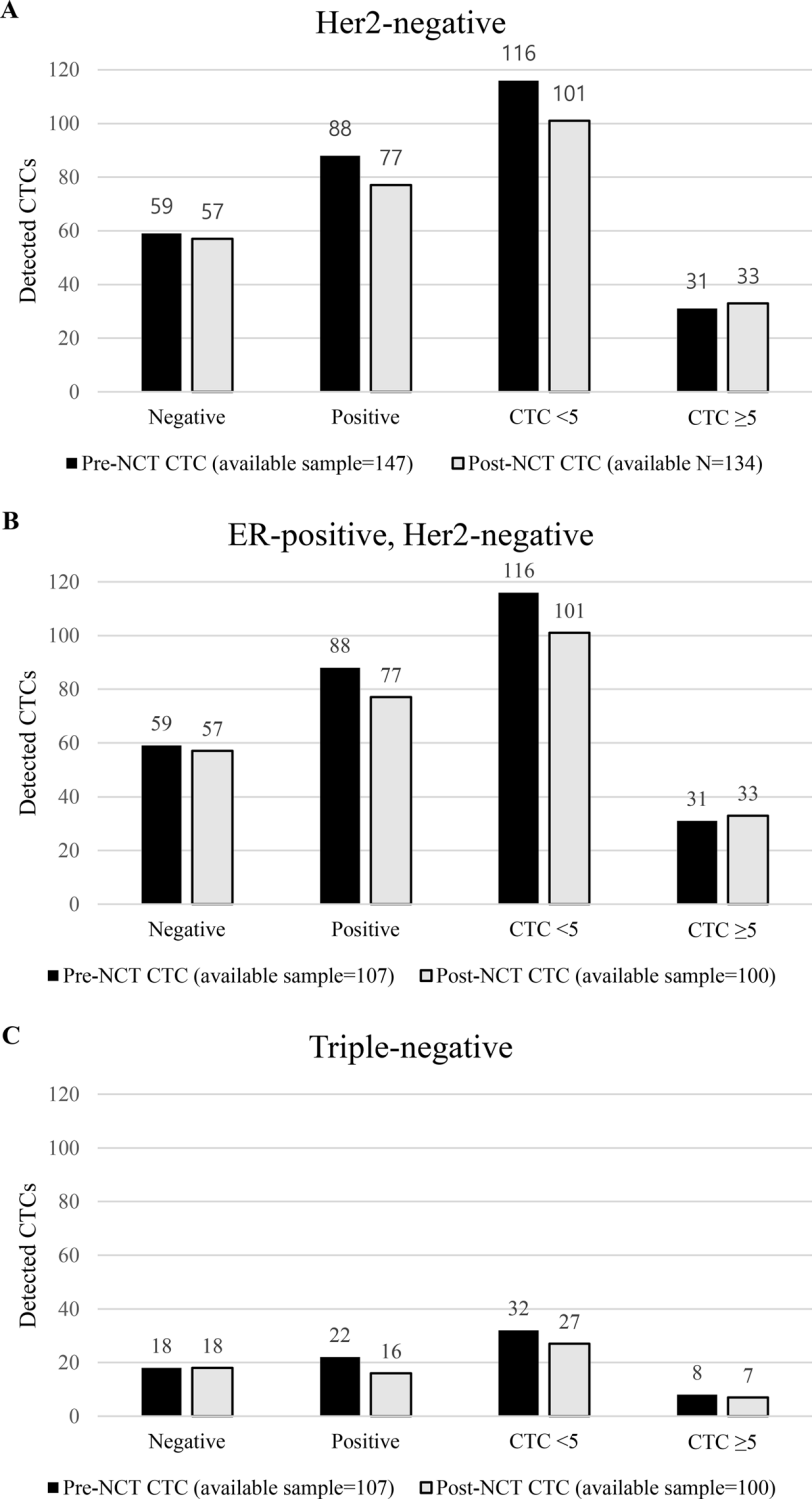
CTC status during the course of NCT. (**A**) Entire Her2-negative cohort, (**B**) ER-positive, Her2-negative group, and (**C**) Triple-negative group. *AC* adriamycin and cyclophosphamide, *CTC* circulating tumor cell, *NCT* neoadjuvant chemotherapy, *pCR* pathologic complete response.

CTC status during the course of NCT of each group were shown in Fig. 1. A total of 147 CTC samples of HER2-negative patients were obtained before NCT and at least one or more CTCs were detected in 88 patients (55.9%; 95% CI, 56.1–71.3%; mean, 2.7 cells; range, 1–18 cells). Post-NCT samples were available for 134 HER2-negative patients, and CTCs were detected in 77 patients (52.4%; 95% CI, 56.1–70.7%; mean, 3.7 cells; range, 1–55 cells). Regardless of time at blood sampling (at baseline, after chemotherapy, changes after NCT), CTC count alone was not associated with the following factors—age, tumor size, nodal status, cancer subtype, nor achievement of pCR (Table 2).

**Table 2:** Cross tabulation analysis of CTC status and pCR status.

Entire HER2-negative patient cohort (N = 147)	pCR		Non-pCR		*X^*2*^	*P*
**Pre-NCT CTC**						
Negative	9	15.3%	50	84.7%	0.831	0.362
Positive	9	10.2%	79	89.8%		
< 5 CTC (0–4)	14	12.1%	102	87.9%	0.016	1.000
≥ 5 CTC	4	12.9%	27	87.1%		
**Post-NCT CTC**						
Negative	6	10.5%	51	89.5%	0.189	0.664
Positive	10	13.0%	67	87.0%		
< 5 CTC (0–4)	13	12.9%	88	87.1%	0.338	0.760
≥ 5 CTC	3	9.1%	30	90.9%		

*CTC* circulating tumor cell, *NCT* neoadjuvant chemotherapy, *pCR* pathologic complete response.

*Chi-square value.

### Patient survival analysis (RFS and OS)

**Figure 2:**
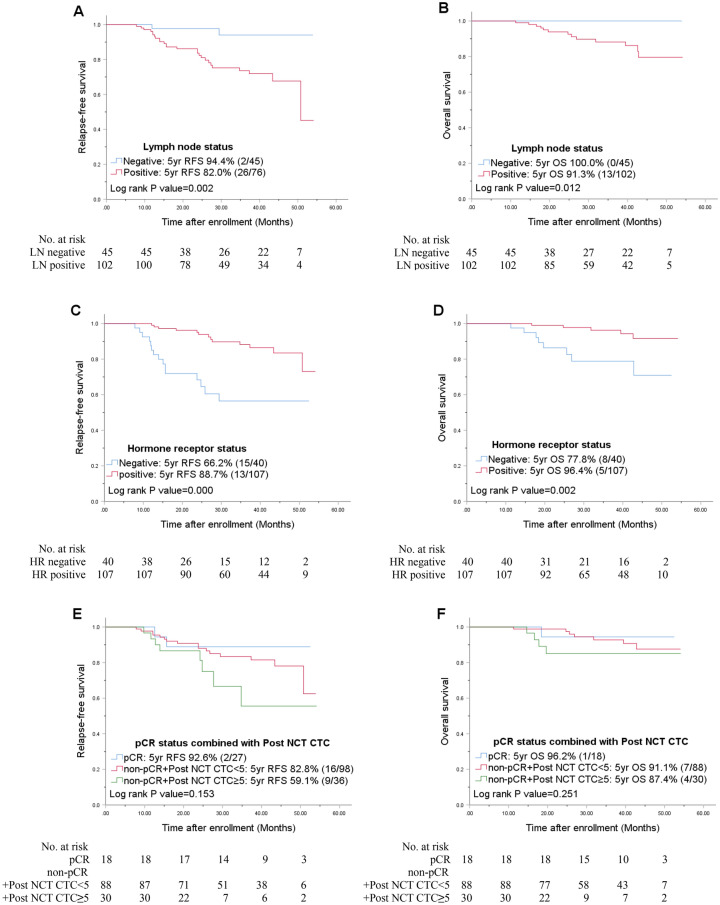
Univariate Kaplan–Meier plots for RFS and OS based on the nodal status (**A,B**), hormone receptor status (**C,D**), and pCR status combined with the post-NCT CTC counts (**E,F**) in the entire HER2-negative cohort. *CTC* circulating tumor cell, *HR* hormone receptor, *LN* lymph node, *NCT* neoadjuvant chemotherapy, *pCR* pathologic complete response, *RFS* relapse-free survival, *OS* overall patient survival.

**Figure 3:**
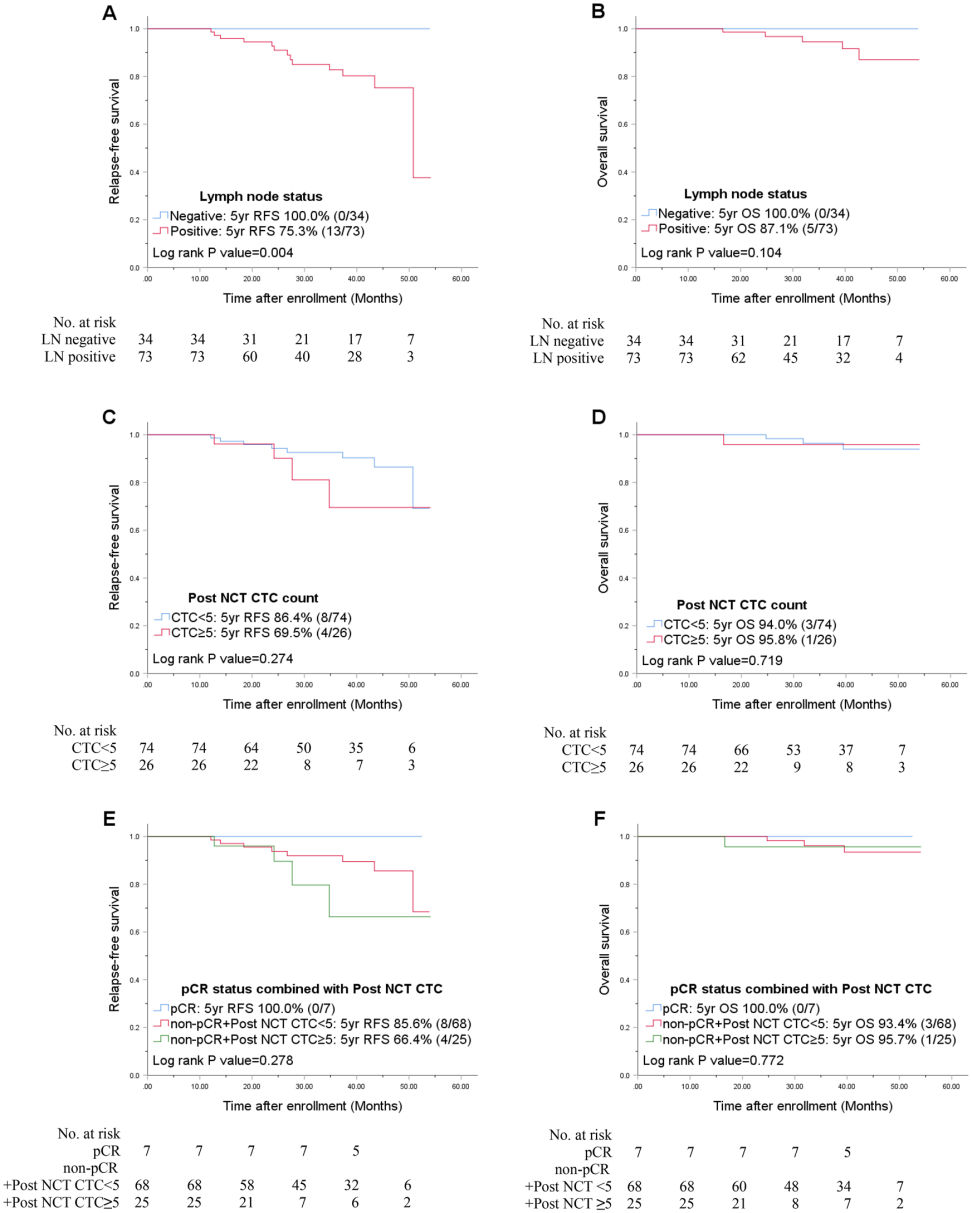
Univariate Kaplan–Meier plots for RFS and OS based on the nodal status (**A,B**), post-NCT CTC counts (**C,D**), and combined with pCR (**E,F**) in the ER-positive, HER2-negative group. *CTC* circulating tumor cell, *HR* hormone receptor, *LN* lymph node, *NCT* neoadjuvant chemotherapy, *pCR* pathologic complete response, *RFS* relapse-free survival, *OS* overall patient survival.

During a median follow-up of 37.3 months, 28 relapses (19.0%) and 13 deaths (8.8%, all breast cancer-specific) were observed among 147 HER2-negative patients. In the entire HER-negative patient cohort (n = 147), the nodal status and hormonal status were statistically significant prognostic factors for both RFS and OS in the univariate analysis (Fig. 2). In the multivariate Cox analysis, the nodal status and hormonal status were consistent risk factors for recurrence (HR, 12.35, 95% CI 1.67–91.83, *p* = 0.014, HR, 8.18, 95% CI 3.47–19.23, *p* = 0.000, respectively) (Table 3). Although the pCR status and post CTC count as independent variables were irrelevant to RFS and OS in univariate analysis (Supplementary Table S1, Supplementary Figure S2 online), when both variables combined, together, it was associated with a worse prognosis for patients who failed to achieve pCR, i.e. the non-pCR group. Furthermore, in the non-pCR group, patients with ≥ 5 CTCs after NCT showed a worse RFS than patients with < 5 CTCs after NCT (HR, 12.86; 95% CI, 2.45–67.43; *p* = 0.003 vs. HR, 4.98; 95% CI, 0.40–27.64; *p* = 0.04) (Table 3). Unlike RFS, only the hormonal status was independently associated with overall patient survival. Compared to the pCR group, the non-pCR group with ≥ 5 CTCs after NCT showed worse OS (HR, 11.19; 95% CI, 1.15–108.71; *p* = 0.037) (Table 3). In the ER-positive, HER2 negative subgroup (n = 107), only the nodal status was relevant to RFS in univariate analysis (*p* = 0.004) (Fig. 3A).

**Table 3:** Univariate and multivariate analyses of RFS and OS in the entire patient cohort and in the triple-negative group.

	RFS			OS		
	Univariate *p*^1^	Multivariate HR (95% CI)^2^	*P*	Univariate *p*^1^	Multivariate HR (95% CI)^2^	*P*
**HER2-negative group (N = 147)**						
Patient age Node negative vs. positive HR positive vs. negative	0.447	0.96 (0.91–1.02)	0.258	0.778	1.00 (0.92–1.09)	0.965
	0.002	12.35 (1.67–91.83)	0.014	0.012	*2.7 × 10^5^	0.962
	0.000	8.18 (3.47–19.23)	0.000	0.002	8.15 (2.31–28.71)	0.001
**PCR vs**						
Non-pCR + post NCT CTC < 5	0.440	4.98 (1.08–23.08)	0.040	0.681	3.31 (0.40–27.64)	0.269
Non-pCR + post NCT CTC ≥ 5	0.102	12.86 (2.45–67.43)	0.003	0.129	11.19 (1.15–108.71)	0.037
**TNBC group (N = 40)**						
Patient age Node negative vs. positive	0.298	0.97 (0.89–1.06)	0.473	0.437	0.96 (0.85–1.08)	0.548
	0.142	4.85 (0.59–39.70)	0.141	0.083	*3.2 × 10^5^	0.975
**PCR vs**						
Non-pCR + post NCT CTC < 5	0.166	3.10 (0.65–14.70)	0.154	0.320	2.90 (0.31–27.43)	0.351
Non-pCR + post NCT CTC ≥ 5	0.001	10.64 (1.80–63.07)	0.009	0.006	14.00 (1.26–155.53)	0.032

*CTC* circulating tumor cell, *HR* hormone receptor, *NCT* neoadjuvant chemotherapy, *OS* overall survival, *pCR* pathologic complete response, *RFS* relapse-free survival, *TNBC* triple-negative breast cancer.

*No events in node negative group.

^1^Log-rank test.

^2^Cox model.

**Figure 4:**
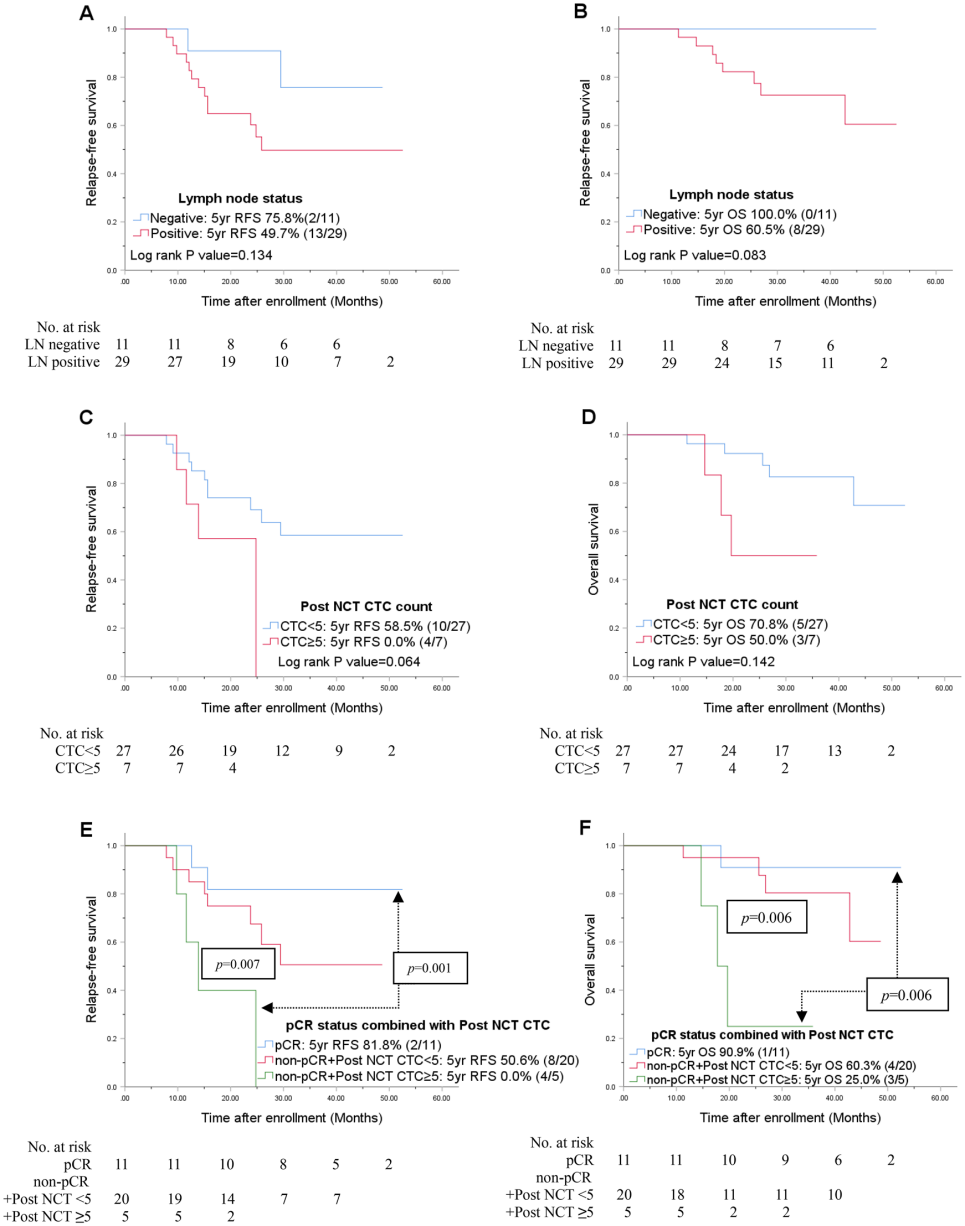
Univariate Kaplan–Meier plots for RFS and OS based on the nodal status (**A,B**), post-NCT CTC counts (**C,D**), and combined with pCR (**E,F**) in the TNBC group. *CTC* circulating tumor cell, *HR* hormone receptor, *LN* lymph node, *NCT* neoadjuvant chemotherapy, *pCR* pathologic complete response, *RFS* relapse-free survival, *OS* overall patient survival.

In the triple-negative subgroup (n = 40), only the post CTC counts showed correlation with OS in univariate analysis *(p* = 0.023, Supplementary Table S1 online), however, when combined with CTC counts after NCT, significant relevance to both RFS and OS were observed. In the non-pCR group, patients with ≥ 5 CTCs after NCT showed worse RFS and OS than patients with < 5 CTCs after NCT (Fig. 4E,F). In multivariate Cox analysis, patients in the non-pCR group with ≥ 5 CTC after NCT showed the worst prognosis in terms of both RFS (HR, 10.66; 95% CI, 1.80–63.07; *p* = 0.009) and OS (HR, 14.00; 95% CI, 1.26–155.53; *p* = 0.032) (Table 3).

## Discussion

In the present study, we analyzed serial CTC counts throughout NCT in locally advanced breast cancer patients, a subset who have been relatively less investigated than metastatic breast cancer patients with evident tumor cells in the circulation. It is well known that pCR has a predictive value in the prognosis in the triple-negative subtype^29,30^. Patients who achieve pCR demonstrate a better prognosis compared to those who fail to achieve pCR^27,31,32^. For those who fail to achieve pCR after NCT, reliable factors that associated with prognosis are relatively rare, especially in terms of their being given additional systemic therapy. Several published studies have demonstrated that the identification of CTCs as a potential biomarker of prognosis after treatment^19,21,33,34^. Hall et al.^23^, showed that CTCs after NCT are related to poor patient prognosis, especially in TNBC. Riethdorf et al.^35^, showed a prognostic association between pCR and pre-NCT CTCs in a neoadjuvant "Geparquattro" trial group. Lucci et al^23^ analyzed CTC count in 57 early-stage TNBC patients after NCT, which is quite similar to our study, and showed one or more CTCs present after NCT predicted relapse and survival in non-metastatic TNBC patients. These findings were similarly observed in our study as 5 CTCs present after NCT correlated with worse survival (*p* = 0.023, Fig. 4). Additionally, we found that among non-pCR TNBC patients, ≥ 5 CTCs after NCT were associated with a worse RFS and OS than < 5 CTCs after NCT, and thus demonstrating an HR of 10.64 and 14.00, respectively, which was not presented by the Lucci’s group. These CTCs after NCT suggest that the burden of residual disease which did not respond to given therapy may eventually cause later relapse or metastasis. Such non-pCR patients demonstrate a 20–30% risk of relapse after NCT (including taxane and anthracycline regimens)^36^; in TNBC patients, particularly, the risk of relapse increases up to 50%^10^. Among these patients, those with hormone receptor-positive tumors have an adjuvant treatment option of an anti-hormonal agent^37^. In this study, patients showed relatively high compliance with adjuvant endocrine therapy (Table 1) compare to conventional reports^38–41^, this may be derived from frequent patient education conducted during the course of treatment and patients’ awareness of the therapeutic benefit of medication and their own recurrence risk^39,42–46^. Masuda et al.^12^ showed the benefit of capecitabine as an adjuvant treatment option for HER2-negative breast cancer patients with residual invasive disease after NCT and currently, the drug is approved for use in TNBC patients not achieving pCR in the U.S.. However, in South Korea, capecitabine is yet to be approved as an adjuvant treatment for TNBC patients fail to achieve pCR after NCT. While CIBOMA/2004-01/GEICAM/2003-11 trial (ClinicalTrials.gov number, NCT00130533)^13^ reported conflicting results than the CREATE-X trial^12^, decisions in real-world are made individual clinicians, implying the necessity of biomarker besides the presence of residual invasive disease. In the present study, we focused on TNBC patients who failed to achieve pCR after NCT. Patients with more than 5 CTCs after NCT displayed a worse outcome. Given that CIBOMA/2004-01/GEICAM/2003-11 trial (ClinicalTrials.gov number, NCT00130533)^13^ fail to show a significant survival benefit of adjuvant capecitabine, except in non-basal subgroup^13^, molecular analysis of CTCs, such as genomic profiling^47,48^ may able to provide useful information that can help find a patient may benefit from the therapy. As patients with the residual disease with no CTCs displayed better outcomes than patients presenting CTCs after NCT, the benefit of additional adjuvant chemotherapy might be profound in those specific groups. Thus, evaluating the clinical utility of the CTC count with subgroup analysis via larger, prospective, randomized neoadjuvant trials to assure its validity may able to show a potential role in tailored therapy.

In metastatic breast cancer, various studies have shown CTC detection to be a potential prognostic factor^16,17,49,50^. However, in non-metastatic breast cancer patients treated with NCT before surgery, the clinical value and prognostic impact of CTCs are less well-investigated^33,51^. Even after curative resection of primary tumors, disseminated tumor cells (DTCs) and micro-metastases can be waiting in ambush as an inactive state for many years^52^ and can recirculate through the bloodstream as CTCs and initiate secondary metastases. Kim et al.^53^ and Leung et al.^54^ suggested that CTCs can give rise to not only distant metastases but also to local relapse, the so-called “tumor self-seeding”. Studies on metastatic breast cancer by Cristofanilli et al.^16^, Nole et al.^55^, and Pierga et al.^56^ suggested 1 CTC/5 ml as a low cut-off value for progression-free survival (PFS) risk, plateauing at 5 CTCs/5 ml. Subsequently, more studies using 1 CTC/5 ml as a cut-off value in non-metastatic breast cancer have been reported^21,22,33,57^. Considering the above studies and the mean CTC counts of this study (pre-NCT, 2.9; post-NCT, 3.9), we applied both the thresholds of 1 CTC/5 ml and 5 CTCs/5 ml after analyzing by several different numbers according to different time-point, subtypes, etc., however, none of them were able to show significant result except cut-off value of 5 CTCs/5 ml.

Currently, most CTC isolation technologies are based on the physical or biological properties of CTCs. The CELLSEARCH system (Veridex LLC, Raritan, NJ, USA) is the only US Food and Drug Administration (FDA)-approved system, which captures CTCs using antibodies directed against EpCAM, and defines CTCs as CK8-positive, CK18-positive or CK19-positive and CD45-negative^58^. However, during the epithelial-mesenchymal transition, EpCAM expression in cancer cells can be decreased as reported by Rao et al.^59^. Thus, this technology may isolate fewer differentiated cancer cells. Sieuwerts, A. M. et al*.*^60^ reported that as there are CTCs without EpCAM expression, EpCAM-based isolation technology may be able to detect a limited number of CTCs. In the current study, although not yet approved for clinical use, we applied both the size and surface antibody technique to overcome this limitation and to isolate both EpCAM-negative and positive CTCs.

Our study has some limitations, the foremost being the lack of validation of the CTC detection method used. The present study was conducted with a CTC detection system by Cytogen, Inc., Seoul, Korea and could not have been validated as the only currently approved CTC detection platform (CELLSEARCH system, Veridex LLC, Raritan, NJ, USA) is not currently available in Korea and even worse, sending samples abroad for analyses was not applicable for the analyses. For this reason, we were not able to perform a direct head-to-head comparison with other studies conducted using the CELLSEARCH system.

. Also, we do not have direct comparison data with the healthy population using this CTC detecting method, that we only included breast cancer patients within this study scope. The sensitivity, specificity and positive predictive values for this method, however, has been previously published in prostate cancer study^61^ with 40.0% sensitivity, 88.2% specificity, 53.2% accuracy, 90.0% PPV, and 35.7% negative predictive value (NPV). Additionally, the assay used in this study cannot detect mesenchymal CTCs, which is a technical limitation of this study. However, the subsequently developed recent version of the system has made it possible to detect mesenchymal CTCs by selecting other epitopes. Another limitation is the relatively short follow-up period to assess survival for prognosis and the missed cases after NCT (13 cases). Since the inclusion criteria of this study regarding patient population and treatment strategies are in a broad range, heterogeneity of these factors also limits the impact of the results of this study (Table 1).

Although the finding of this study showed that patients who fail to achieve pCR and ≥ 5 CTCs after NCT displayed the worst outcome, this does not support routine clinical use of this specific assay used in this study, due to limitations addressed above. However, the findings could provide justification to launch a future, well designed trial with longer follow-up data to obtain regulatory approval for clinical use of the assay, especially for the ER-positive, HER2-negative breast cancer subset.

## Materials and methods

### Patients and treatments

Eligibility criteria for the study were female gender with age > 20 years and with NCT and no distant metastases. From February 2014 to May 2017, excluding patients with metastatic disease detected by systemic work-up (whole body PET scan/chest CT scan/abdominal and pelvic CT scan/bone scan), 173 non-metastatic breast cancer patients treated with NCT at Asan Medical Center in Seoul, Korea were enrolled in the present study. CTC detection was done before and after NCT, i.e. after two cycles for a total of four cycles of the chemotherapy regimen and after four cycles for a total of eight cycles of the chemotherapy regimen. After NCT, 13 patients were eliminated from our study due to patient decisions such as their refusal to undergo blood sampling after NCT. Neither the patients nor the clinicians were informed of the CTC results. From the entire population of 173 patients, we excluded 26 HER2-positive patients as the given neoadjuvant anti-HER2 regimen was heterogeneous and which could affect both the pCR rate and the outcome. The study was approved by the Institutional Review Board of Asan Medical Center (Seoul, Korea) (IRB-e no. 2013–1048), and was compliant with the REMARK criteria^62^. Written informed consent was obtained from all of the enrolled patients. All methods were performed in accordance with the relevant guidelines and regulations.

The initial diagnostic and follow-up work-up included mammography, breast ultrasound imaging, magnetic resonance imaging, chest X-rays, blood sampling, and clinical examination. Estrogen receptor and progesterone receptor expression were evaluated based on the Allred score ^63^. The HER2 status was confirmed as negative if the immunohistochemistry score was 1 + , or if the score was 2 + and the result of fluorescence or silver in situ hybridization for HER2 amplification was negative^64^. All of the clinical and histopathological staging was based on the 7th edition of the Cancer Staging Manual of the American Joint Committee on Cancer^65^.

All of the patients with hormone receptor-positive tumors subsequently received adjuvant tamoxifen or aromatase inhibitors after surgery.

### Circulating tumor cell detection—blood collection and CTC enrichment

A volume of 5 cc of blood was collected from each patient into Acid Citrate Dextrose tubes (BD Vacutainer; BD Biosciences, San Jose, CA, USA) and the samples were processed within four hours to minimize cell loss and processing failure^47,66–68^. CTC isolation was performed using a SMART BIOPSY SYSTEM Isolation kit (cat no. CIKW10; Cytogen, Inc., Seoul, Korea)^61,66,67^. Briefly, blood samples were incubated with 20 µg/µl of antibody cocktail (complex) from the SMART BIOPSY SYSTEM Isolation kit (Cytogen, Inc.) targets white blood cells (CD45) and red blood cells (globin), captures epithelial CTCs, which was uniquely developed by Cytogen Inc., for 20 min and were then mixed with pre-activation buffer followed by density gradient centrifugation at 400 × g for 30 min at room temperature. The cell suspension containing CTCs was collected and gradually diluted with dilution buffer (Cytogen, Inc.). Diluted cell suspensions were filtered through an HDM chip (Cytogen, Inc.), as previously described^69^. Cells on the HDM chip were collected and transferred to a microtube. For immunofluorescence staining, isolated cells were fixed on slides in 4% paraformaldehyde for five minutes at room temperature and were then kept at 4 °C until further processing^70^.

### Circulating tumor cell detection—immunofluorescence staining

**Figure 5:**
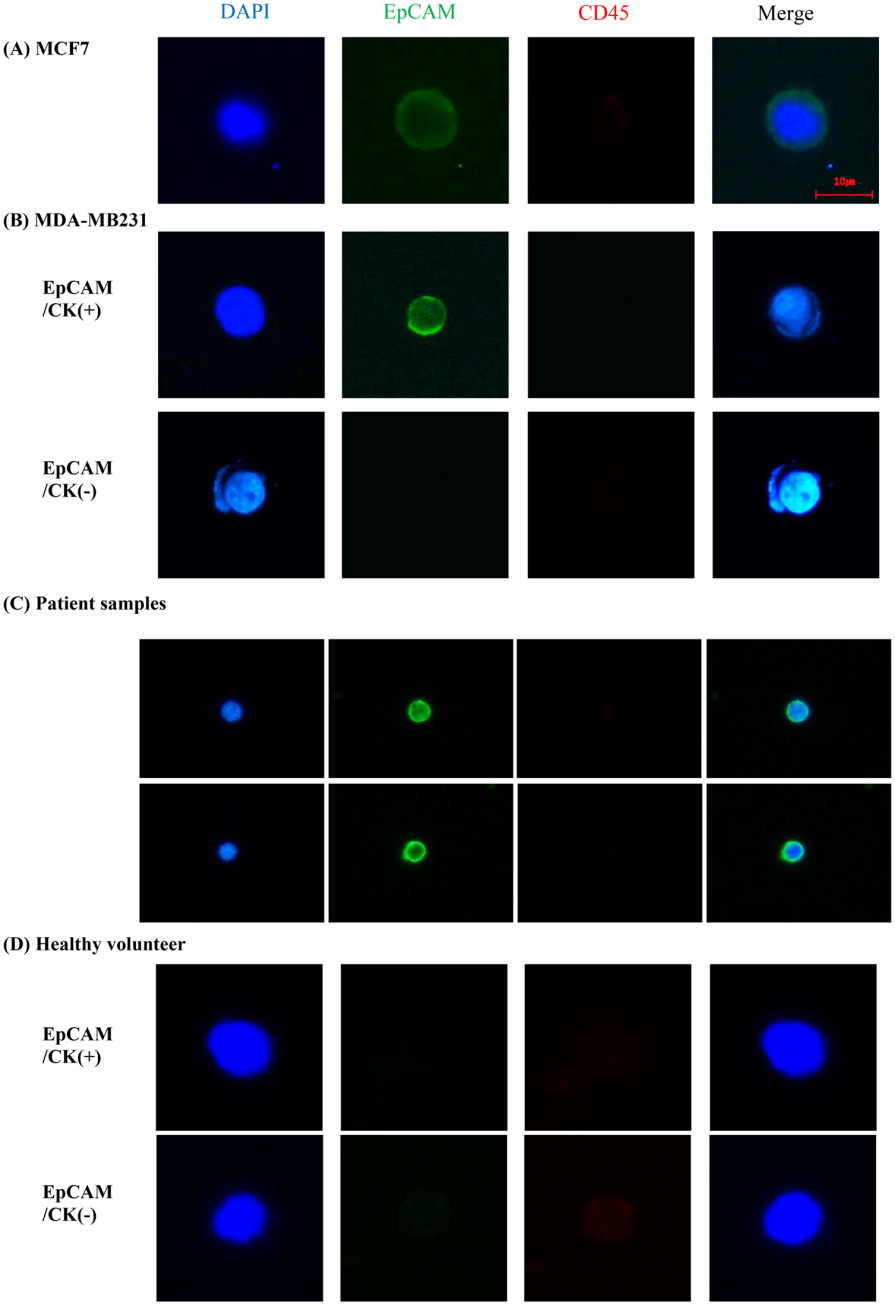
Immunofluorescence image of EpCAM-positive and CD45-negative CTC in MCF-7 (**A**), MDA-MB231 (**B**), patient samples (**C**), and healthy volunteer (**D**). *CD45* cluster of differentiation 45, *CK* cytokeratin, *CTC* circulating tumor cell, *DAPI* 4′,6-diamidino-2-phenylindole, *EpCAM* epithelial cell adhesion molecule, *MCF7* Michigan Cancer Foundation-7, *MDA-MB231* MD. Anderson-metastatic breast 231.

The MCF7 human breast cancer cell line was used for positive control. Cells on slides were permeabilized with 0.2% Triton X-100 in PBS for 10 min at room temperature. Cells were then blocked with 1% bovine serum albumin in PBS for 60 min and incubated with primary antibodies for 60 min, followed by secondary antibody incubation under the same conditions. The primary antibodies used were mouse anti-EpCAM (Cell Signaling Technology), mouse anti-cytokeratin (Sigma), and rabbit anti-CD45 (Cell Signaling Technology). The secondary antibody used was goat anti-rabbit Alexa Fluor 647 (Thermo Fisher Scientific, Inc.) and goat anti-mouse Alexa Fluor 488 (Thermo Fisher Scientific, Inc.). The slides were mounted using Fluoroshield Mounting Medium with DAPI (ImmunoBioScience Corp). Stained cells were observed and images captured using a fluorescence microscope (Eclipse Ti; Nikon Corporation, Tokyo, Japan) with a 400× objective^70^ (Fig. 5). Quantification was done by a human observer. And one slide was processed per patient.

### Spike‑in test with MCF7 and MDA-MB231 cells for confirmation of CTC capture efficiency

A total of 100 breast cancer cell lines (MCF7 or MDA-MB231) were spiked into 1 ml of healthy volunteers' blood, which underwent the same CTC isolation protocol described above using SMART BIOPSY SYSTEM Isolation kit (cat no. CIKW10; Cytogen, Inc., Seoul, Korea). The isolated cell suspension was transferred to a new dish and the number of EpCAM( +) and CD45(-) cells were counted under a fluorescent microscope (Eclipse Ti; Nikon Corporation) within 30 min. Experiments were performed in triplicate. The Cell recovery rate was determined as follows: Cell recovery rate (%) = (No. of detected cells / total input cells) × 100. (Supplmentary Figure S1).

### Treatment response and survival analysis

Treatment response evaluations were performed by physical examination and using imaging assessments, at baseline, after the first administration of treatment and at the completed course of NCT. Tumor responses evaluation was abided by the Response Evaluation Criteria In Solid Tumors (RECIST 1.1)^26,71^. In every treatment phase, physical examinations with serologic tests were performed. Relapse-free survival (RFS) and overall survival (OS) were evaluated from a detailed review of the electronic medical records (EMR) data. All of the patients received standard treatment, and surveillance was performed according to their physicians’ decisions.

### Statistical methods

RFS and OS were analyzed in the entire HER2-negative population as well as within each subgroup, i.e. ER-positive with HER2-negative group and TNBC group. RFS was defined as the time from the date of the study enrollment to the first date of disease recurrence, and OS was defined as the time from the date of the study enrollment to the date of a patient’s death from any cause. The probability of patient survival was estimated using the Kaplan–Meier method and Cox regression analysis. Multivariate Cox proportional hazards regression analyses were performed using the following clinical parameters: patient age at the time of diagnosis; clinical tumor stage; lymph node status; hormone receptor status; and HER2 positivity. All statistical tests were conducted using IBM SPSS Statistics version 26.0 for Windows (SPSS, Inc./IBM Co.), and a value of *p* < 0.05 was considered statistically significant.

## Supplementary information

Supplementary Table S1.

Supplementary Figure S1.

Supplementary Figure S2.

Supplementary Figure S3.
