# Publisher Correction: Analysis of the serial circulating tumor cell count during neoadjuvant chemotherapy in breast cancer patients

**DOI:** 10.1038/s41598-021-85731-3

**Published:** 2021-03-11

**Authors:** Sungchan Gwark, Jisun Kim, Nak-Jung Kwon, Kyoung-Yeon Kim, YongNam Kim, Cham Han Lee, Young Hun Kim, Myoung Shin Kim, Sung Woo Hong, Mi Young Choi, Byung Hee Jeon, Suhwan Chang, Jonghan Yu, Ji Yeon Park, Hee Jin Lee, Sae Byul Lee, Il Yong Chung, Beom Seok Ko, Hee Jeong Kim, Jong Won Lee, Byung Ho Son, Jin-Hee Ahn, Kyung Hae Jung, Sung-Bae Kim, Gyung-Yub Gong, Sei Hyun Ahn

**Affiliations:** 1 Department of Surgery, University of Ulsan, College of Medicine, Asan Medical Center, Seoul, Korea; 2 Macrogen Inc, Seoul, Korea; 3 Cytogen Inc, Seoul, Korea; 4 Department of Biomedical Sciences, University of Ulsan, College of Medicine, Asan Medical Center, Seoul, Korea; 5 Department of Surgery, Division of Breast and Endocrine Surgery, Sungkyunkwan University School of Medicine, Samsung Medical Center, Seoul, Korea; 6 Department of Pathology, University of Ulsan, College of Medicine, Asan Medical Center,, Seoul, Korea; 7 Department of Oncology, University of Ulsan, College of Medicine, Asan Medical Center,, Seoul, Korea

Correction to: *Scientific Reports* 10.1038/s41598-020-74577-w, published online 15 October 2020

The original version of this Article contained an error in the order of author names, which was incorrectly given as Sung-chan Gwark, Jisun Kim, Cham Han Lee, Young Hun Kim, Myoung Shin Kim, Sung Woo Hong, Mi Young Choi, Byung Hee Jeon, Nak‑Jung Kwon, Kyoung‑Yeon Kim, Yongnam Kim, Suhwan Chang, Jong‑Han Yu, Ji Yeon Park, Jin-Hee Ahn, Kyung Hae Jung, Sung‑Bae Kim, Hee Jin Lee, Gyung‑Yub Gong, Sae Byul Lee, Il Yong Chung, Beom Seok Ko, Hee Jeong Kim, Jong Won Lee, Byung Ho Son & Sei Hyun Ahn.

Furthermore, the original version of this Article contained errors in the spelling of the authors Sungchan Gwark, YongNam Kim and Jonghan Yu, which were incorrectly given as Sung-chan Gwark, Yongnam Kim and Jong‑Han Yu respectively.

These errors have now been corrected in the HTML and PDF versions of the Article, and in the accompanying Supplementary Information files.

