# Supplementary material for: Analysis of the serial circulating tumor cell count during neoadjuvant chemotherapy in breast cancer patients
Source: Sci Rep. 2020 Oct 15;10:17466. doi: 10.1038/s41598-020-74577-w (PMC7562710; doi:10.1038/s41598-020-74577-w)
Supplement: Supplementary file 1 — Supplementary Table S1. [file 41598_2020_74577_MOESM1_ESM.docx]

**Analysis of the serial circulating tumor cell count during neoadjuvant chemotherapy in breast cancer patients**

**Sungchan Gwark^1^, Jisun Kim^1^, Nak-Jung Kwon^2^, Kyoung-Yeon Kim^2^, YongNam Kim^2^, Cham Han Lee^3^, Young Hun Kim^3^, Myoung Shin Kim^3^, Sung Woo Hong^3^, Mi Young Choi^3^, Byung Hee Jeon^3^, Suhwan Chang^4^, Jonghan Yu^5^, Ji Yeon Park^1^, Hee Jin Lee^6^, Sae Byul Lee^1^, Il Yong Chung^1^, Beom Seok Ko^1^, Hee Jeong Kim^1^, Jong Won Lee^1^, Byung Ho Son^1^, Jin-Hee Ahn^7^, Kyung Hae Jung^7^, Sung-Bae Kim^7^,** [**Gyung-Yu**](https://www.liebertpub.com/doi/10.1089/thy.2017.0334)**b Gong^6^, Sei Hyun Ahn^1^**

^1^Department of Surgery, University of Ulsan, College of Medicine, Asan Medical Center, Seoul, Korea.

^2^Macrogen Inc, Seoul, Korea.

^3^Cytogen Inc, Seoul, Korea.

^4^Department of Biomedical Sciences, University of Ulsan, College of Medicine, Asan Medical Center, Seoul, Korea.

^5^Department of Surgery, Division of Breast and Endocrine Surgery, Sungkyunkwan University School of Medicine, Samsung Medical Center, Seoul, Korea.

^6^Department of Pathology, University of Ulsan, College of Medicine, Asan Medical Center, Seoul, Korea.

^7^Department of Oncology, University of Ulsan, College of Medicine, Asan Medical Center, Seoul, Korea.

* Correspondence and requests for materials should be addressed to J.K (email: [jisunkim@amc.seoul.kr](mailto:jisunkim@amc.seoul.kr))

| ***HER2-negative group***  (N=147) | **RFS** | | | **OS** | | |
| --- | --- | --- | --- | --- | --- | --- |
|  | **Univariate *p*^1^** | **Multivariate HR**  **(95% CI)^2^** | ***P*** | **Univariate *p*^1^** | **Multivariate HR**  **(95% CI)^2^** | ***P*** |
| **Patient age**  **Node negative vs. positive**  **HR positive vs. negative**  **pCR vs. non-pCR**  **Post NCT CTC <5 vs. ≥5** | 0.447 | 0.97 (0.91–1.02) | 0.240 | 0.778 | 1.00 (0.92–1.09) | 0.955 |
|  | 0.002 | 12.81 (1.73–94.91) | 0.013 | 0.012 | *2.7 x 10^5^ | 0.962 |
|  | 0.000 | 8.05 (3.43–19.92) | 0.000 | 0.002 | 8.20 (2.32–28.98) | 0.001 |
|  | 0.292 | 4.48 (0.98-20.52) | 0.053 | 0.497 | 3.16 (0.39-25.42) | 0.280 |
|  | 0.169 | 2.53 (1.06-6.07) | 0.036 | 0.181 | 3.16 (0.89-11.18) | 0.074 |
| ***TNBC group***  (N=40) |  | | | | | |
| **Patient age**  **Node negative vs. positive**  **pCR vs. non-pCR**  **Post NCT CTC <5 vs. ≥5** | 0.298 | 0.96 (0.88–1.05) | 0.377 | 0.437 | 0.95 (0.85–1.07) | 0.399 |
|  | 0.142 | 5.75 (0.74–44.88) | 0.095 | 0.083 | *3.4 x 10^5^ | 0.974 |
|  | 0.087 | 2.90 (0.63-13.31) | 0.170 | 0.184 | 2.69 (0.32-22.90) | 0.365 |
|  | 0.064 | 2.86 (0.84-9.74) | 0.093 | 0.023 | 4.24 (0.89-20.11) | 0.069 |

**No events in node negative group*

**Supplement Table S1.** Univariate and multivariate analyses of RFS and OS in the entire patient cohort and in the triple-negative group. pCR status and CTC status as as independent variables. Abbreviation: CTC, circulating tumor cell; HR, hormone receptor; NCT, neoadjuvant chemotherapy; OS, overall survival; pCR, pathologic complete response; RFS, relapse-free survival; TNBC, triple-negative breast cancer. ^1^Log-rank test; ^2^Cox model
