# Supplementary material for: Analysis of the serial circulating tumor cell count during neoadjuvant chemotherapy in breast cancer patients
Source: Sci Rep. 2020 Oct 15;10:17466. doi: 10.1038/s41598-020-74577-w (PMC7562710; doi:10.1038/s41598-020-74577-w)
Supplement: Supplementary file 2 — Supplementary Figure S1. [file 41598_2020_74577_MOESM2_ESM.docx]

**Analysis of the serial circulating tumor cell count during neoadjuvant chemotherapy in breast cancer patients**

**Sungchan Gwark^1^, Jisun Kim^1^, Nak-Jung Kwon^2^, Kyoung-Yeon Kim^2^, YongNam Kim^2^, Cham Han Lee^3^, Young Hun Kim^3^, Myoung Shin Kim^3^, Sung Woo Hong^3^, Mi Young Choi^3^, Byung Hee Jeon^3^, Suhwan Chang^4^, Jonghan Yu^5^, Ji Yeon Park^1^, Hee Jin Lee^6^, Sae Byul Lee^1^, Il Yong Chung^1^, Beom Seok Ko^1^, Hee Jeong Kim^1^, Jong Won Lee^1^, Byung Ho Son^1^, Jin-Hee Ahn^7^, Kyung Hae Jung^7^, Sung-Bae Kim^7^,** [**Gyung-Yu**](https://www.liebertpub.com/doi/10.1089/thy.2017.0334)**b Gong^6^, Sei Hyun Ahn^1^**

^1^Department of Surgery, University of Ulsan, College of Medicine, Asan Medical Center, Seoul, Korea.

^2^Macrogen Inc, Seoul, Korea.

^3^Cytogen Inc, Seoul, Korea.

^4^Department of Biomedical Sciences, University of Ulsan, College of Medicine, Asan Medical Center, Seoul, Korea.

^5^Department of Surgery, Division of Breast and Endocrine Surgery, Sungkyunkwan University School of Medicine, Samsung Medical Center, Seoul, Korea.

^6^Department of Pathology, University of Ulsan, College of Medicine, Asan Medical Center, Seoul, Korea.

^7^Department of Oncology, University of Ulsan, College of Medicine, Asan Medical Center, Seoul, Korea.

* Correspondence and requests for materials should be addressed to J.K (email: [jisunkim@amc.seoul.kr](mailto:jisunkim@amc.seoul.kr))

|  | **Retrieval rate (%)** | **Retrieval mean (%)** | **CV** |
| --- | --- | --- | --- |
| **MCF7** | 69.07 | 70.19 | 5.51 |
|  | 67.00 |  |  |
|  | 74.49 |  |  |
| **MDA-MB231** | 65.77 | 60.08 | 3.43 |
|  | 58.62 |  |  |
|  | 61.54 |  |  |

**B**

**A**

**Supplementary Figure S1.** Efficiency of breast cancer cell isolation following the spike-in test using healthy donor blood. Experiments were performed in triplicate. (A) Retrieval rate of each experiment and mean retrieval rate. (B) Recovery rate following the spike-in test. MCF7 (epithelial cell), MDA-MB231 (mesenchymal cell) were spiked into peripheral blood samples from healthy donors (100 cells each). CV, coefficient of variation; MCF7, Michigan Cancer Foundation-7; MDA-MB231, M.D. Anderson-Metastatic Breast cancer cell 231.
