# Supplementary material for: Analysis of the serial circulating tumor cell count during neoadjuvant chemotherapy in breast cancer patients
Source: Sci Rep. 2020 Oct 15;10:17466. doi: 10.1038/s41598-020-74577-w (PMC7562710; doi:10.1038/s41598-020-74577-w)
Supplement: Supplementary file 3 — Supplementary Figure S2. [file 41598_2020_74577_MOESM3_ESM.docx]

**Analysis of the serial circulating tumor cell count during neoadjuvant chemotherapy in breast cancer patients**

**Sungchan Gwark^1^, Jisun Kim^1^, Nak-Jung Kwon^2^, Kyoung-Yeon Kim^2^, YongNam Kim^2^, Cham Han Lee^3^, Young Hun Kim^3^, Myoung Shin Kim^3^, Sung Woo Hong^3^, Mi Young Choi^3^, Byung Hee Jeon^3^, Suhwan Chang^4^, Jonghan Yu^5^, Ji Yeon Park^1^, Hee Jin Lee^6^, Sae Byul Lee^1^, Il Yong Chung^1^, Beom Seok Ko^1^, Hee Jeong Kim^1^, Jong Won Lee^1^, Byung Ho Son^1^, Jin-Hee Ahn^7^, Kyung Hae Jung^7^, Sung-Bae Kim^7^,** [**Gyung-Yu**](https://www.liebertpub.com/doi/10.1089/thy.2017.0334)**b Gong^6^, Sei Hyun Ahn^1^**

^1^Department of Surgery, University of Ulsan, College of Medicine, Asan Medical Center, Seoul, Korea.

^2^Macrogen Inc, Seoul, Korea.

^3^Cytogen Inc, Seoul, Korea.

^4^Department of Biomedical Sciences, University of Ulsan, College of Medicine, Asan Medical Center, Seoul, Korea.

^5^Department of Surgery, Division of Breast and Endocrine Surgery, Sungkyunkwan University School of Medicine, Samsung Medical Center, Seoul, Korea.

^6^Department of Pathology, University of Ulsan, College of Medicine, Asan Medical Center, Seoul, Korea.

^7^Department of Oncology, University of Ulsan, College of Medicine, Asan Medical Center, Seoul, Korea.

* Correspondence and requests for materials should be addressed to J.K (email: [jisunkim@amc.seoul.kr](mailto:jisunkim@amc.seoul.kr))


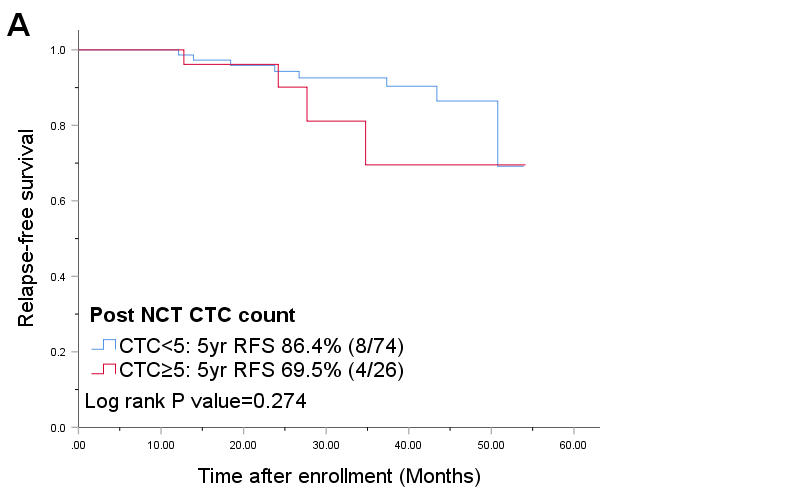

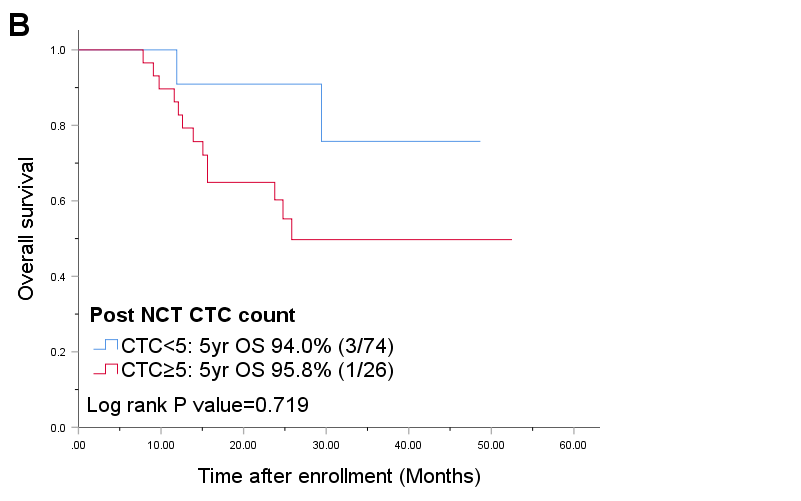


No. at risk

CTC<5 101 101 89 69 49 8 1

CTC≥5 33 33 25 10 8 3 1

No. at risk

CTC<5 101 100 82 61 43 7 1

CTC≥5 33 33 25 8 7 3 1


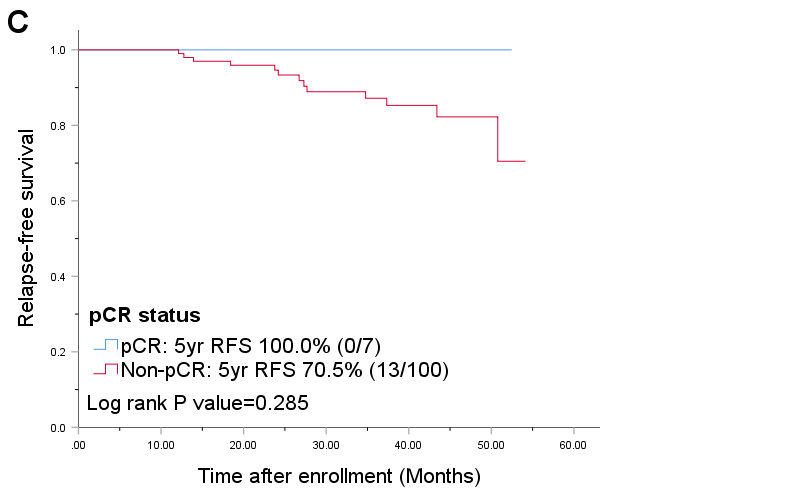

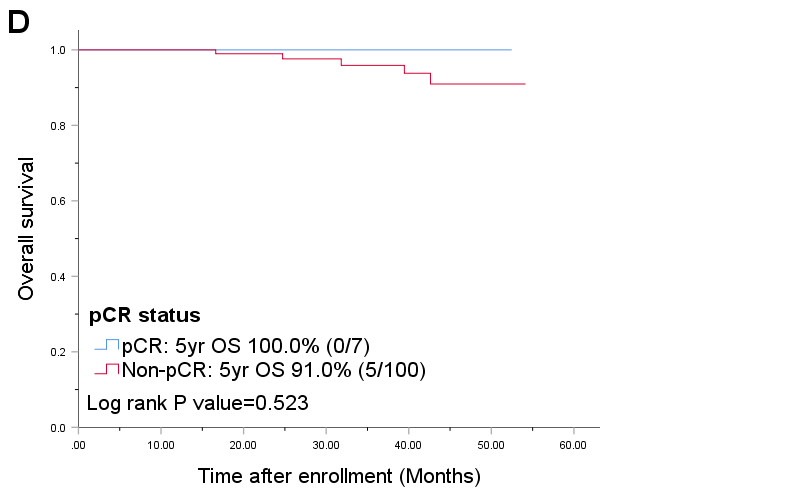


No. at risk

pCR 7 7 7 7 5 2 2

non-pCR 100 100 83 54 40 8 1

No. at risk

pCR 7 7 7 7 5 2 2

non-pCR 100 100 85 59 44 9 1

**Supplementary Figure S2.** Univariate Kaplan–Meier plots for RFS and OS based on post-NCT CTC counts (**A, B)** and on pCR status (**C, D**) in the **entire HER2-negative cohort.** Abbreviation: CTC, circulating tumor cell; HR, hormone receptor; LN, lymph node; NCT, neoadjuvant chemotherapy; pCR, pathologic complete response; RFS, relapse-free survival; OS, overall patient survival.
