# Supplementary material for: Analysis of the serial circulating tumor cell count during neoadjuvant chemotherapy in breast cancer patients
Source: Sci Rep. 2020 Oct 15;10:17466. doi: 10.1038/s41598-020-74577-w (PMC7562710; doi:10.1038/s41598-020-74577-w)
Supplement: Supplementary file 4 — Supplementary Figure S3. [file 41598_2020_74577_MOESM4_ESM.docx]

**Analysis of the serial circulating tumor cell count during neoadjuvant chemotherapy in breast cancer patients**

**Sungchan Gwark^1^, Jisun Kim^1^, Nak-Jung Kwon^2^, Kyoung-Yeon Kim^2^, YongNam Kim^2^, Cham Han Lee^3^, Young Hun Kim^3^, Myoung Shin Kim^3^, Sung Woo Hong^3^, Mi Young Choi^3^, Byung Hee Jeon^3^, Suhwan Chang^4^, Jonghan Yu^5^, Ji Yeon Park^1^, Hee Jin Lee^6^, Sae Byul Lee^1^, Il Yong Chung^1^, Beom Seok Ko^1^, Hee Jeong Kim^1^, Jong Won Lee^1^, Byung Ho Son^1^, Jin-Hee Ahn^7^, Kyung Hae Jung^7^, Sung-Bae Kim^7^,** [**Gyung-Yu**](https://www.liebertpub.com/doi/10.1089/thy.2017.0334)**b Gong^6^, Sei Hyun Ahn^1^**

^1^Department of Surgery, University of Ulsan, College of Medicine, Asan Medical Center, Seoul, Korea.

^2^Macrogen Inc, Seoul, Korea.

^3^Cytogen Inc, Seoul, Korea.

^4^Department of Biomedical Sciences, University of Ulsan, College of Medicine, Asan Medical Center, Seoul, Korea.

^5^Department of Surgery, Division of Breast and Endocrine Surgery, Sungkyunkwan University School of Medicine, Samsung Medical Center, Seoul, Korea.

^6^Department of Pathology, University of Ulsan, College of Medicine, Asan Medical Center, Seoul, Korea.

^7^Department of Oncology, University of Ulsan, College of Medicine, Asan Medical Center, Seoul, Korea.

* Correspondence and requests for materials should be addressed to J.K (email: [jisunkim@amc.seoul.kr](mailto:jisunkim@amc.seoul.kr))

**H1 (No CTC detected)**


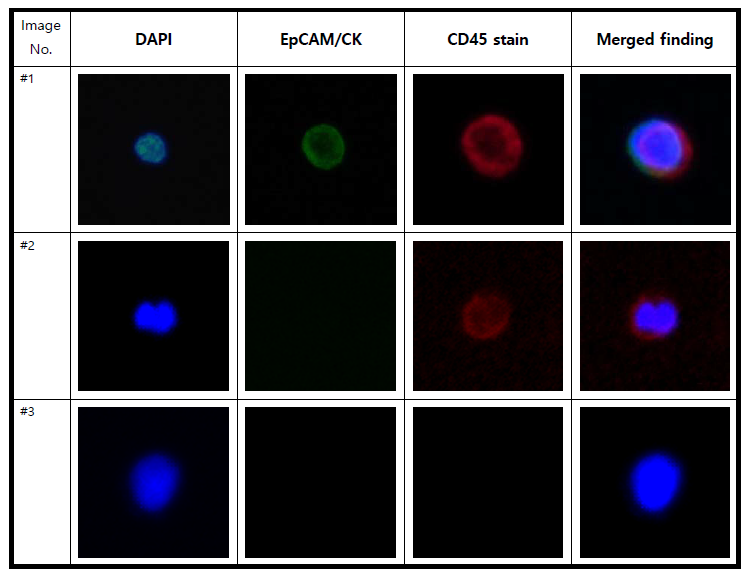


**H2 (No CTC detected)**


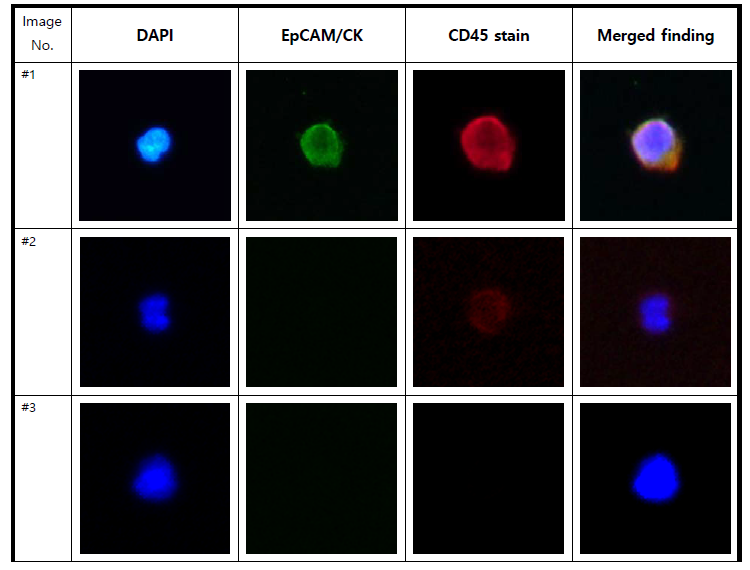


**H3 (No CTC detected)**


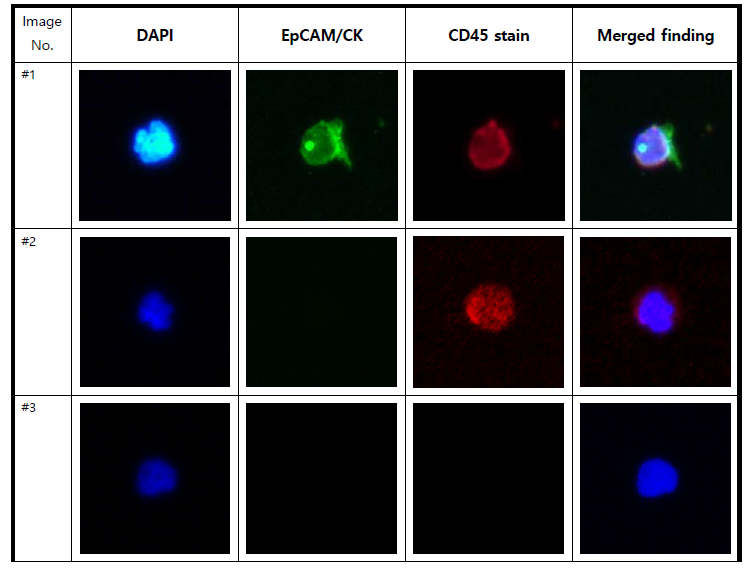


**H4 (No CTC detected)**


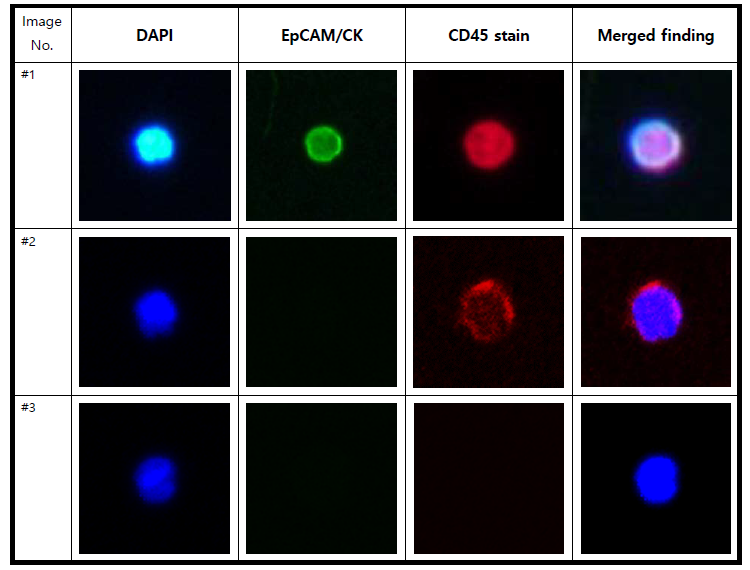


**H5 (No CTC detected)**


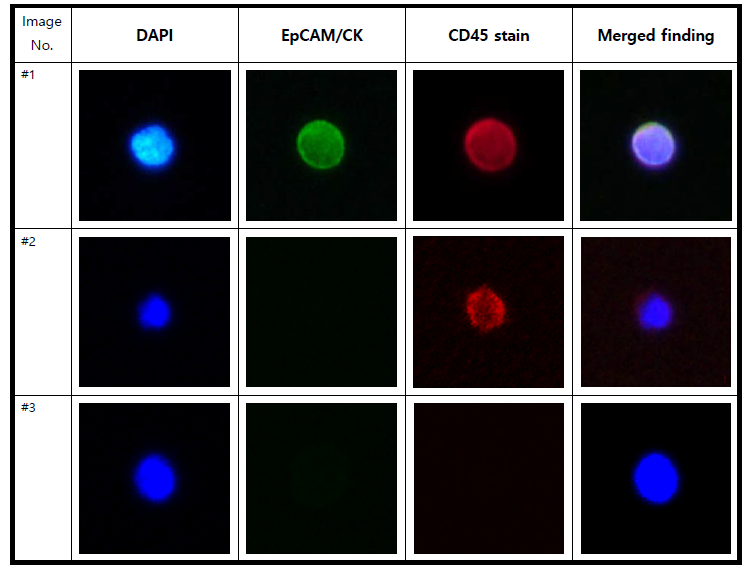


**H6 (1 CTC detected)**


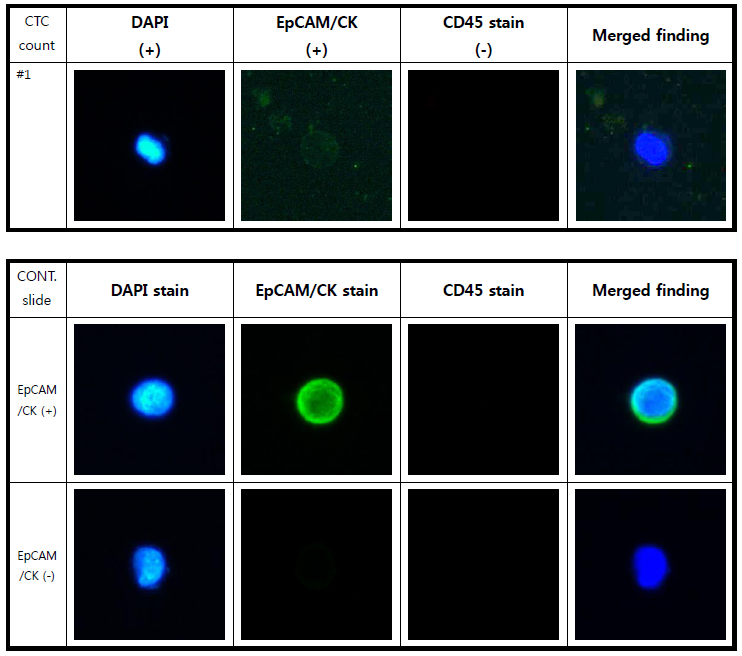


**H7 (1 CTC detected)**


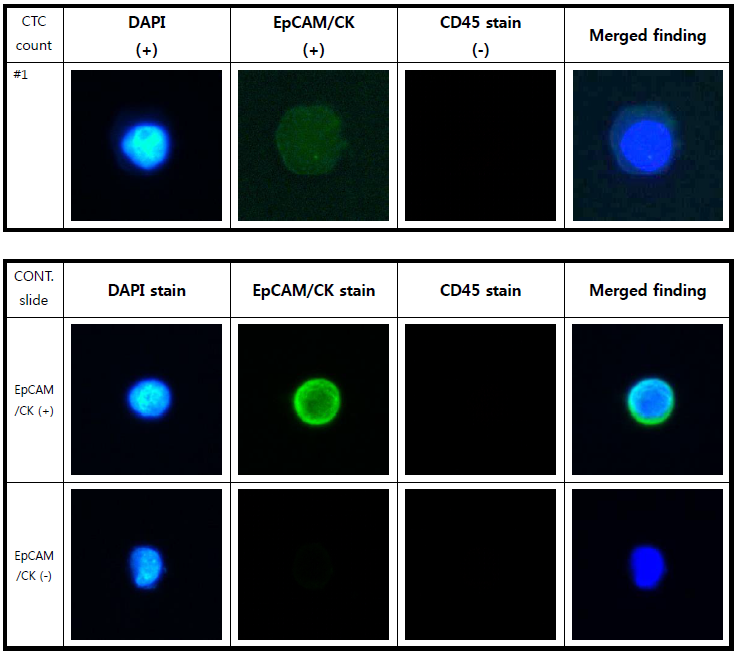


**H8 (3 CTCs detected)**


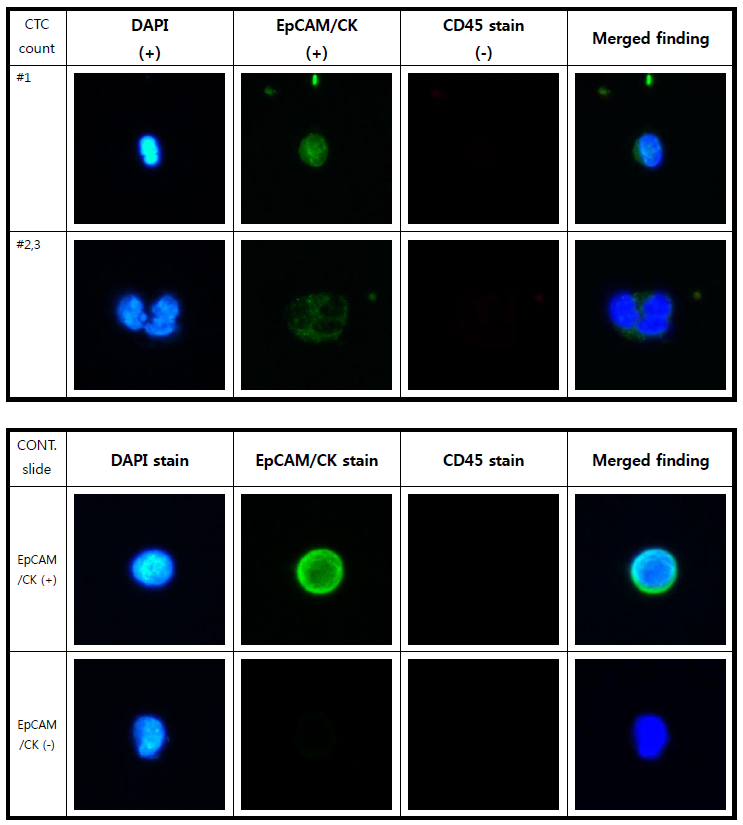


**H9 (No CTC detected)**


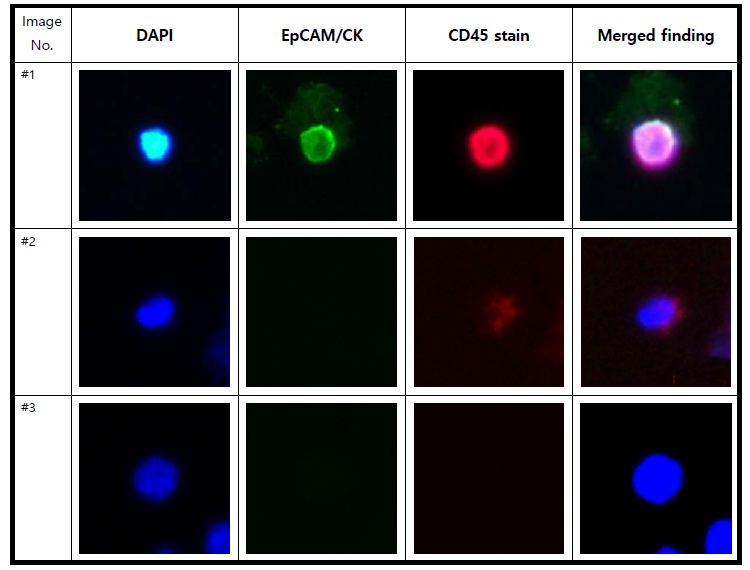


**H10 (No CTC detected)**


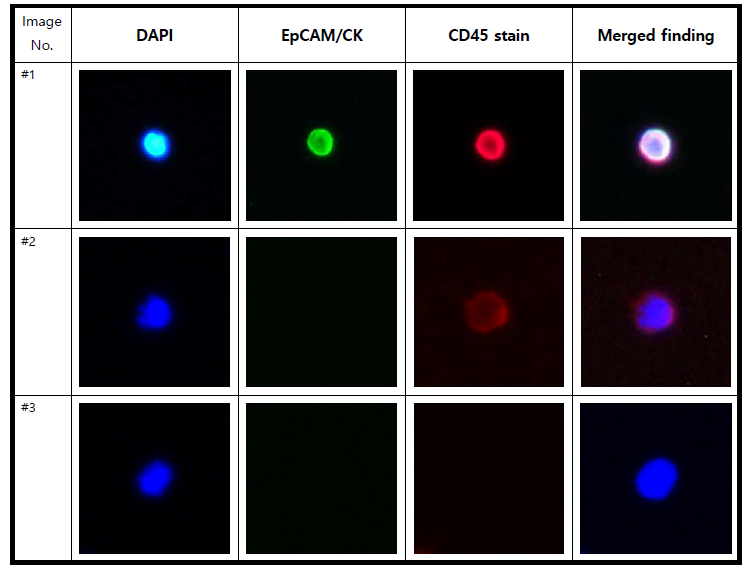


**H11 (No CTC detected)**


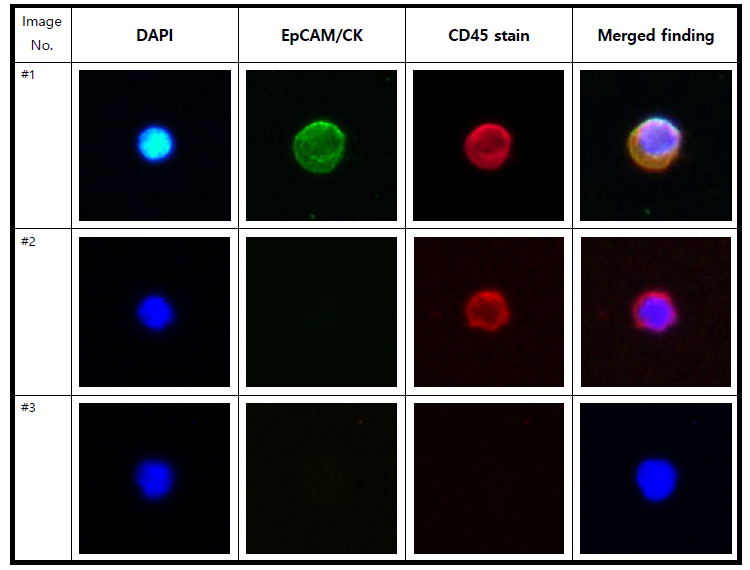


**H12 (No CTC detected)**


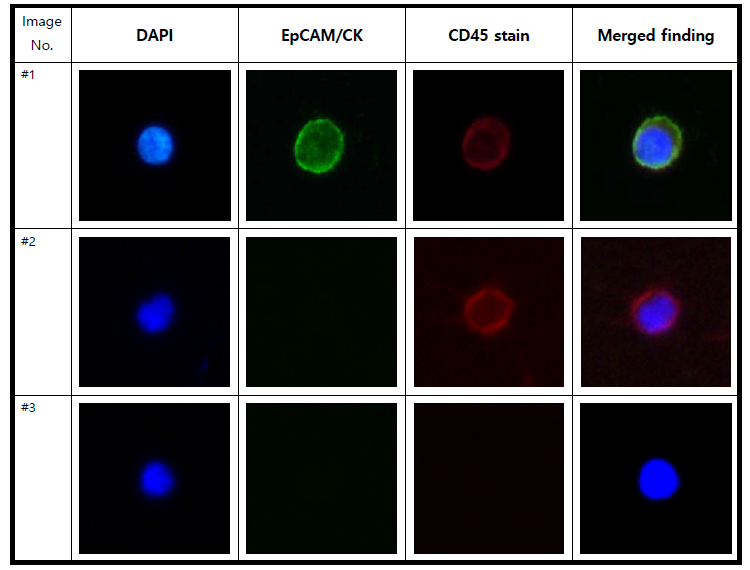


**H13 (No CTC detected)**


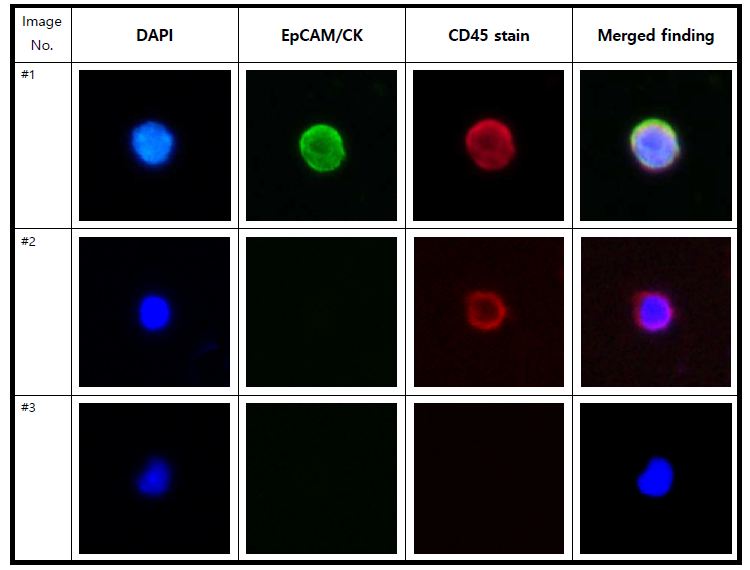


**H14 (No CTC detected)**


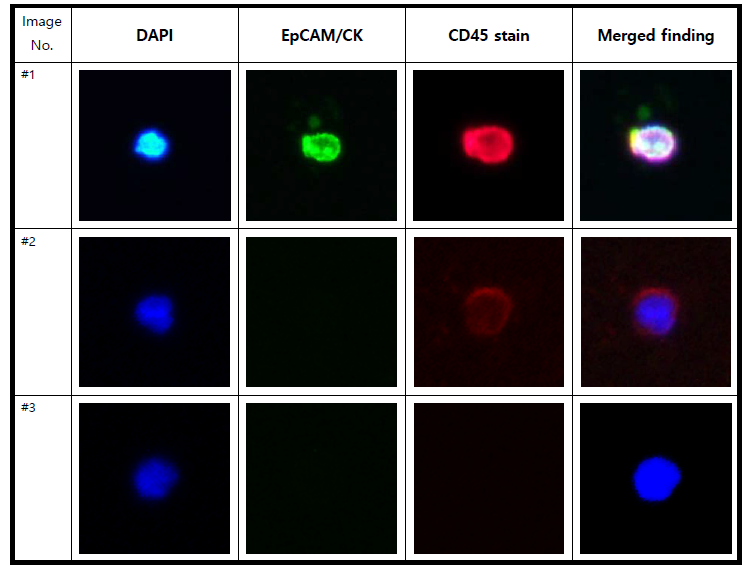


**H15 (No CTC detected)**


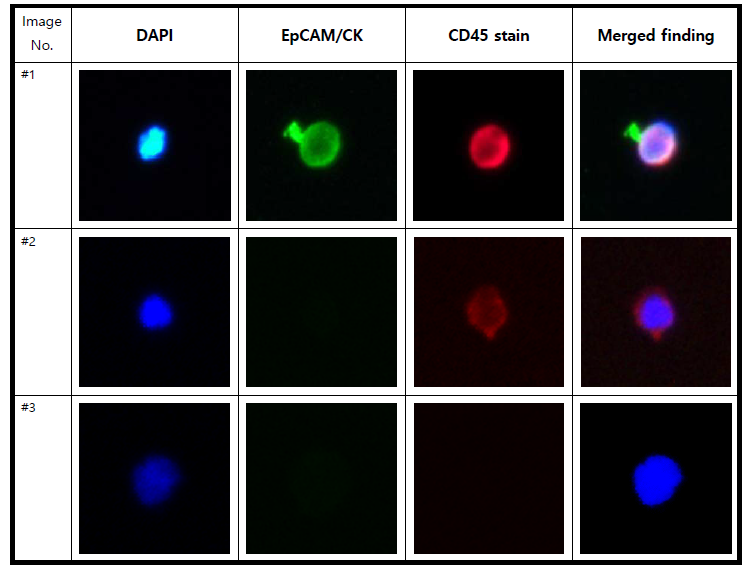


**Supplementary Figure S3.** Immunofluorescence image of healthy volunteers (H1-H15). Abbreviation: CD45, cluster of differentiation 45; CK, cytokeratin; CTC, circulating tumor cell; DAPI, 4′, 6-diamidino-2-phenylindole; EpCAM, epithelial cell adhesion molecule.
